# Pathognomonic Symptom of Cerebro-Spinal Meningitis

**Published:** 1876-09

**Authors:** 


PATHOGNOMONIC SYMPTOM OF CEREBRO-SPINAL MENINGITIS.—Dr. Hayden, of Dublin, a competent authority, states that he never saw a case of cerebro-spinal meningitis unattended by pains in the calves of the legs, and he should make a presumptive diagnosis from the presence of that symptom alone.—*Med. and Surg. Reporter.*

